# Impact of stopping contact precautions on *vanA* plasmid transmission, Northern California, 2021–2023

**DOI:** 10.1017/ash.2026.10392

**Published:** 2026-05-12

**Authors:** Guillermo Rodriguez-Nava, Matthew P. Grieshop, Alessandro Zulli, Aaron A. Behr, Eugenia Miranti, Wajeeha Tariq, Erika Paola Viana-Cardenas, Nathan Pincus, Niaz Banaei, Mindy Sampson, Ami S. Bhatt, Jorge L. Salinas

**Affiliations:** 1 Stanford University School of Medicine, USA; 2 Denver Health and Hospital Authority, USA; 3 University of Colorado Anschutz Medical Campus School of Medicine, USA; 4 Stanford University Department of Civil and Environmental Engineering, USA

## Abstract

**Objective:**

To evaluate the impact of discontinuing contact precautions for vancomycin-resistant enterococci (VRE) on strain and plasmid transmission using long-read whole-genome sequencing (WGS).

**Study design:**

Before-after trial of adults with *Enterococcus* bloodstream infections pre-(Jan–Oct 2021) and post-(Oct–Dec 2021 and Jan–Oct 2023) discontinuation of contact precautions for VRE infections.

**Setting:**

Quaternary referral and transplant academic medical center.

**Patients:**

Hospitalized adults (≥18 yr) with *E. faecalis* or *E. faecium* bacteremia.

**Methods:**

Classical epidemiology identified potential transmissions via shared unit exposure within a 14-day window. Blood culture isolates underwent long-read WGS to assess strain and *vanA* plasmid relatedness. Clonal transmission was defined as <20 single-nucleotide polymorphisms. Plasmid similarity was assessed with Mash distance.

**Findings:**

Among 288 isolates from 202 patients, there was no significant difference in possible epidemiologic transmissions pre-versus postdiscontinuation (9.5% vs 8.1%; *P* = .679). Genomic analysis identified four clonal transmission events, two of which occurred postdiscontinuation. Among 70 *vanA* plasmids from 54 patients, 38 highly related plasmids formed a low-diversity cluster. The proportion of cluster plasmids was not significantly different between periods (47% vs 60%; *P* = .267). Postdiscontinuation, *vanA*-positive *E. faecium* ST117 was more prevalent (22/44 vs 53/75; *P* = .024).

**Conclusion:**

Discontinuation of contact precautions for VRE was not associated with increased transmission of enterococci or *vanA* plasmids in bloodstream infections. Transmission patterns remained largely stable, though the postdiscontinuation period showed increased prevalence of the *dominant E. faecium* ST117. These findings suggest limited impact of contact precautions on VRE transmission.

## Introduction

Enterococci are commonly implicated in healthcare-associated infections (HAIs) and approximately 30% of these isolates are vancomycin-resistant enterococci (VRE).^1^ Vancomycin resistance is typically mediated by the *vanA* gene cluster which alters the glycopeptide binding site and is often carried by MGEs that enable inter-strain and inter-species transmission.^2^ Following the emergence of vancomycin resistance in the late 1980s, clonality studies supported the idea that VRE spreads primarily through person-to-person transmission in health-care settings.^3–5^ For this reason, and to limit the spread of vancomycin resistance, the Hospital Infection Control Practices Advisory Committee recommended contact precautions (single room, gowns, and gloves) for patients with VRE infections in 1995.^3^

The effectiveness of contact precautions in preventing vancomycin resistance spread remains debated, with limited high-quality evidence. A robust randomized controlled trial would require 50 clusters or 25 centers per group, making it impractical.^6^ Consequently, most evidence comes from before-and-after studies, where hospitals discontinuing contact precautions reported no significant increases in VRE infections.^7^ Whole-genome sequencing (WGS) offers a cost-effective adjunct, providing detailed transmission insights without large trials and enabling precise tracking of bacterial spread. Previous studies used short-read WGS to evaluate VRE transmission before and after discontinuing contact precautions.^8,9^ However, short-read methods may underestimate MGE-mediated resistance spread due to difficulty resolving repetitive regions and linking resistance genes to their carriers. Long-read WGS enables more complete assembly of bacterial and plasmid genomes, allowing precise tracking of MGE-borne resistance and its genomic context.^10^ We used long-read WGS for a more detailed analysis of the transmission dynamics of the *vanA* plasmid following discontinuation of contact precautions for VRE bloodstream infections.

## Methods

### Study population, epidemiologic design, and data collection

This study was conducted at Stanford University Medical Centre, a quaternary referral and transplant center with 800 combined licensed beds. On October 1, 2021, Stanford Health Care discontinued the routine use of contact precautions for patients with VRE infections. Specifically, single-room placement, gowns, and gloves were no longer required for patients with active VRE-positive clinical cultures across the hospital. Colonization was not systematically tracked. We conducted a retrospective, quasi-experimental study integrating classical epidemiologic methods and WGS to assess the impact of contact precaution discontinuation on VRE strain and plasmid transmission. No other hospitalwide infection prevention interventions were introduced or discontinued during the study periods. No VRE outbreaks were identified during either study period.

We included all blood culture isolates yielding *E. faecalis* or *E. faecium* from hospitalized adults (≥18 yr) collected during two periods: prediscontinuation (January 1–September 30, 2021) and postdiscontinuation (October 1–December 31, 2021, and January 1–October 31, 2023). We included vancomycin-resistant and-susceptible isolates to capture extensive genomic diversity of circulating *Enterococcus faecalis* and *E. faecium* in our hospital. Isolates from 2022 were unavailable, as the clinical microbiology laboratory retains bloodstream infection isolates for one year. We retrospectively collected patient metadata, including demographics, clinical characteristics, and immunocompromised status—defined as active chemotherapy, hematologic malignancy, solid organ transplant, or prednisone ≥20 mg/day (or equivalent). Additional data included vancomycin exposure within the prior three months, culture results, susceptibility profiles, and location tracking across all units available through the electronic medical record, including wards, ICUs, emergency department, operative suites, catheterization and interventional radiology laboratories, and procedural areas. Only radiology units were excluded, as specific imaging locations cannot be automatically extracted from the medical record. Potential transmissions were identified epidemiologically among patients with VRE bacteremia, based on shared-location exposure within seven days before or after the index positive blood culture collection date, consistent with an estimated VRE colonization incubation period of approximately 5–9 days.^11–13^ The study was approved by the Stanford University Institutional Review Board.

### Isolate collection, whole-genome sequencing, and genomic analysis

Bloodstream *E. faecalis* and *E. faecium* isolates were identified by MALDI-TOF (Bruker Biotyper). Frozen stocks were cultured aerobically for 16 hours in 3 mL brain heart infusion (BHI) broth at 37°C with shaking. A 1:100 subculture was then incubated until reaching OD_600_ ∼ 1.0. Cell pellets were resuspended in PBS, centrifuged, and stabilized in Zymo DNA/RNA Shield™ before shipment to Plasmidsaurus (Oregon, USA) for long-read sequencing using Oxford Nanopore Technology (ONT) with DNA extraction. For genome assembly and bioinformatics analysis, DNA libraries were sequenced with ONT using the “Standard Bacterial Genome with DNA extraction” protocol. Assemblies were generated by the sequencing provider and assessed for taxonomic accuracy and contiguity (Supplementary Data). Assemblies with more than 20 contigs were excluded. Additional details on isolate collection, library preparation, and sequencing have been published elsewhere.^14^

Chromosomal and plasmid contigs were classified using geNomad (v1.8.0), a bioinformatics tool combines machine learning with reference-based methods for element identification.^15^ Contigs with scores >0.6 (chromosome) or >0.7 (plasmid) were retained; others were excluded due to low classification confidence, often reflecting ambiguous elements such as potential phage or unresolved plasmid sequences. Pairwise chromosomal and plasmid distances were estimated using Mash (v2.3), a tool that rapidly approximates genome-wide similarity. Mash uses MinHash, a probabilistic algorithm that compresses large sequences into representative sketches, enabling fast comparison and clustering of closely related genomes.^16^ To assess genomic relatedness and identify potential strain transmission events, split *k*-mer analysis (SKA v1.0) was applied pairwise to all chromosomal contigs. Split k-mer analysis identifies shared *k-mers* across samples separated by a single base and is ideally suited for clinical outbreak tracking.^17^ A conservative threshold of 20 single-nucleotide polymorphisms (SNPs) was used to define clonal transmission, based on comparisons of isolates from the same patients.

### Statistical analysis

Group comparisons were performed using the Mann–Whitney *U* test for continuous variables. For proportions, the *Z*-test or Fisher’s exact test was used, as appropriate. A *P* value of <.05 was considered statistically significant.

## Results

### Baseline characteristics of the study cohort

We collected 288 blood culture isolates in total. Of these, 105 (36.2%) were from the prediscontinuation period and 183 (63.8%) from the postdiscontinuation period, representing 202 unique patients. Compared to the prediscontinuation group, the postdiscontinuation group had a higher proportion of immunocompromised patients (24.1% vs 39.1%, *P* = .007) and a longer length of stay (17 vs 23 d, *P* = .004). There were no significant differences in age, sex, surgical or oncological status, or hospital-onset bacteremias, defined as onset on day 4 or later of admission. Vancomycin-resistant *E. faecalis* was more common in the postdiscontinuation group (0% vs 6.4%, *P* = .044), while vancomycin-resistant *E. faecium* was not significantly different between pre-and postdiscontinuation groups (72.7% versus 69.3%, *P* = .392) (Table 1).

**Table 1.** Patient characteristics and microbiologic data of patients with *Enterococcus* bloodstream infection during pre- and post-discontinuation of contact precautions periods—Northern California, 2021–2023

	Prediscontinuation	Postdiscontinuation	*P* value*
Isolates (%)	105 (36.5)	183 (63.5)	
Age (range) years	66 (23–102)	67 (19–99)	.324
Sex: female (%)	34 (32.4)	53 (29)	.543
Immunocompromised (%)	26 (24.8)	72 (39.3)	.011*
Oncological (%)	21 (20)	44 (24)	.429
Surgical (%)	25 (23.8)	58 (31.7)	.155
LOS days (range)	16 (1–475)	23 (1–387)	.007*
Hospital onset bacteremia (%)	46 (43.8)	83 (45.4)	.799
Vancomycin exposure (%)	55 (52.4)	111 (60.7)	.171
Vancomycin days of therapy	2 (1–33)	3 (1–78)	.725
*E. faecalis* (%)	61 (58.1)	108 (59)	.878
Vancomycin-resistant (%)	0 (0)	6 (5.6)	.060
Vancomycin exposure (%)	23 (37.7)	53 (49.1)	.153
Vancomycin days of therapy	3 (1–33)	2 (1–78)	.676
*E. faecium* (%)	44 (41.9)	75 (41)	.153
Vancomycin-resistant (%)	32 (72.7)	52 (69.3)	.694
Vancomycin exposure (%)	32 (72.7)	58 (77.3)	.572
Vancomycin days of therapy	2 (1–27)	3 (1–32)	.253

Comparisons between groups were performed using the Mann–Whitney *U* test for continuous variables and the *Z*-test for proportions. *p < .05.

LOS, length of stay.

### Strain transmission dynamics postdiscontinuation of contact precautions

During the study period, 850 potential encounters were identified where patients specifically with VRE bloodstream infection shared the same unit within the defined ±7 day window period. The most common shared locations were the operating room (324 pairs), transplant step-down unit (192), emergency department (132), general medical ward (64), and surgical ICU (58). After excluding duplicate encounters beyond the initial shared-location event, 25 unique encounter pairs remained. The proportion of potential transmissions based on classical epidemiology was similar before (9.52%, 95% CI: 3.91%–15.14%) and after (8.2%, 95% CI: 4.22%–12.17%) the discontinuation of contact precautions (difference 1.33%, 95% CI: –8.21% to 5.55%; *P* = .700). In sensitivity analyses extending the shared-location window from ±7 days to ±14 and ±21 days around the index blood culture, the proportion of potential epidemiologic transmissions remained similar or lower postdiscontinuation (±14-day window: 24.8% vs 15.3%, *P* = .048; ±21-day window: 41.9% vs 22.4%, *P* < .001), with higher proportions observed in the prediscontinuation period in both analyses.

**Figure 1.**
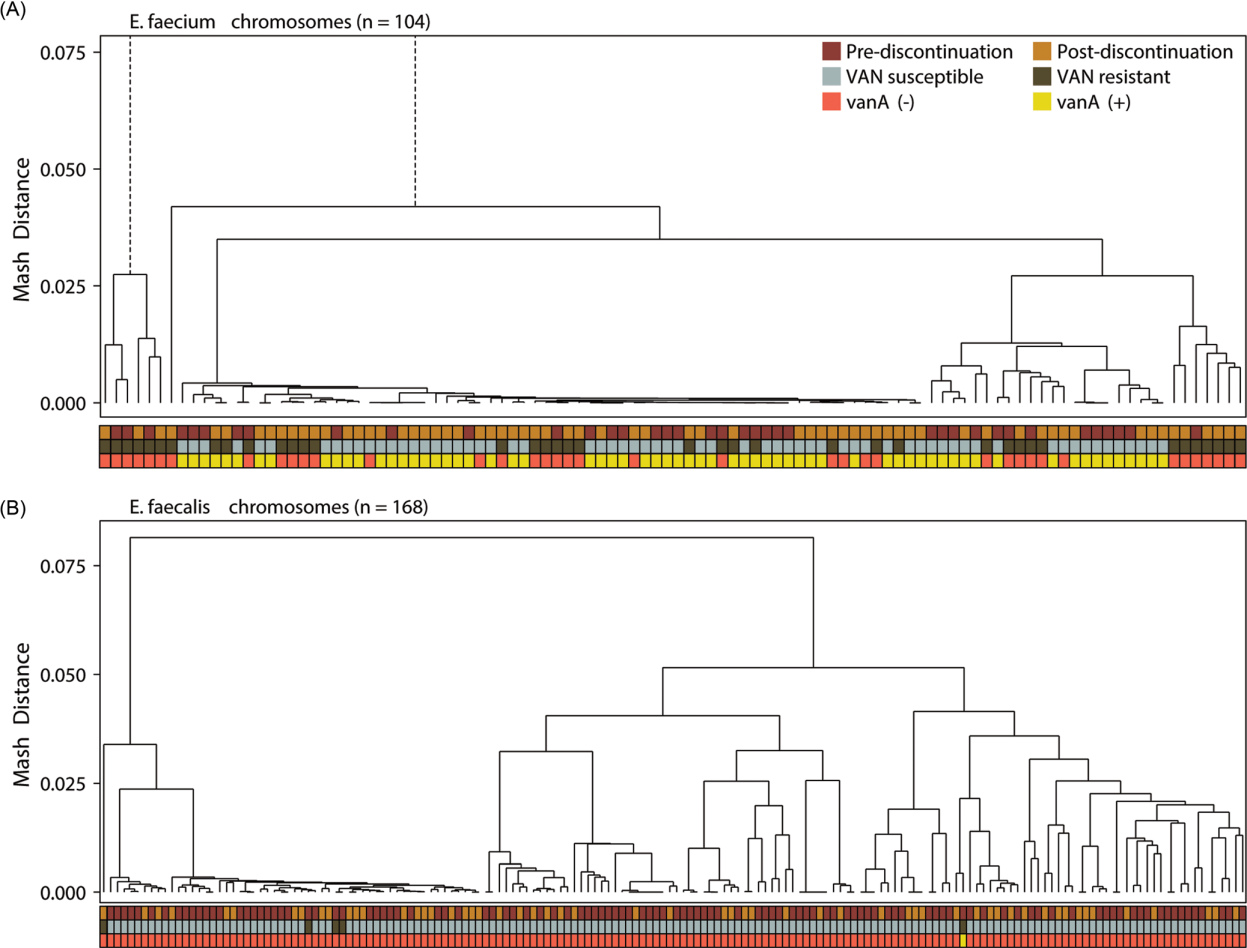
Chromosomal Mash distance clustering of *Enterococcus* isolates from Stanford Health Care, 2021–2023. All panels display hierarchical clustering of pairwise Mash distances calculated from whole-genome sketches (k-mer 21); branch lengths reflect genetic divergence. In A, *E. faecium* (n = 104); in B, *E. faecalis* (n = 168). Beneath each tree, three annotation tracks denote, from top to bottom, timing of obtention relative to discontinuation of contact precautions (Red Oxide = prediscontinuation; Golden Ocher = postdiscontinuation), vancomycin susceptibility phenotype (Ash Gray = susceptible; Dark Khaki = resistant), and *vanA* plasmid presence (Coral Red = absent; Citrine Yellow = present).

A total of 266 highly contiguous genome assemblies were generated using long-read WGS, including 168 *E. faecalis* and 104 *E. faecium* genomes. Isolates initially identified as Enterococcus but reclassified as non-Enterococcus by sequencing and those failing assembly quality thresholds were excluded. Chromosomal and plasmid contigs were identified using geNomad, and genomic distances were estimated with Mash. These data were paired with metadata on phenotypic vancomycin resistance and *vanA* plasmid presence. The resulting dendrograms for *E. faecium* and *E. faecalis* are shown in Figure 1A,B.

**Figure 2.**
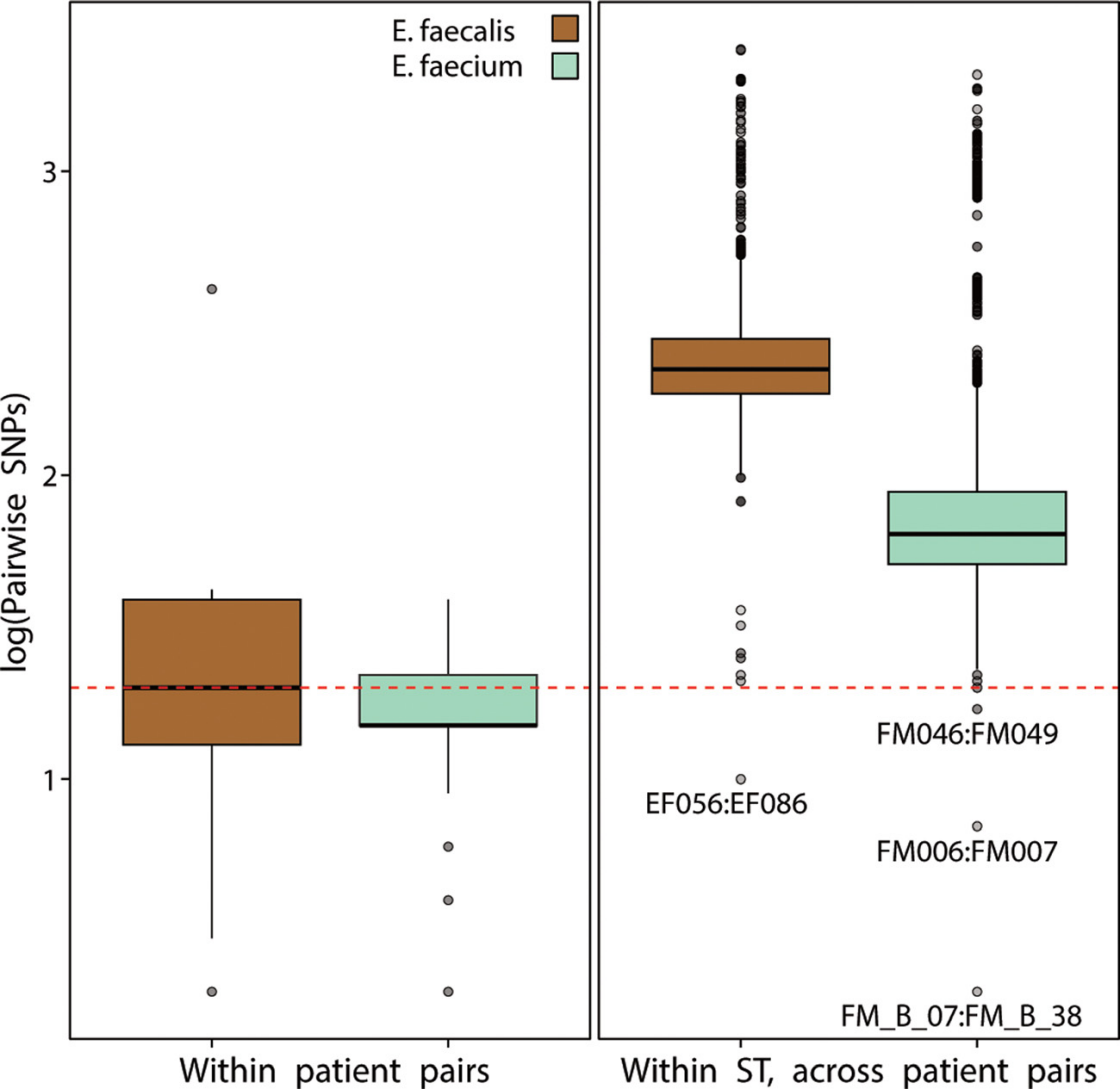
Comparison of pairwise chromosomal SNP distances between *Enterococcus* isolates, Stanford Health Care, 2021–2023. All panels display boxplots of log_10_(pairwise SNP counts): left, within patient isolate pairs; right, within the same sequence type (ST) across different patients. Boxes show median and interquartile range; whiskers extend to 1.5×IQR and points beyond are outliers. Colors denote species (Golden Brown *= E. faecalis*; Seafoam Green = *E. faecium*). The red dashed line indicates 20 SNPs, the cutoff for inferring clonal relatedness (values at or below represent putative transmission events); selected pairings near or below that threshold are annotated.

To explore whether putative transmission events of VRE strains increased postdiscontinuation of contact precautions, split k-mer analysis was applied pairwise to all chromosomal contigs to identify SNP sites. The threshold for putative transmission events was derived from within-patient isolate pairs, assuming they represented the same strain, and was set at 20 or fewer SNPs based on split *k*-mer analysis (Figure 2). Four cross-patient isolate pairs met the genomic threshold suggesting potential strain sharing, fewer than those identified by classical epidemiology: three involved *E. faecium* and one involved *E. faecalis* (Figure 2). Among the *E. faecium* pairs, two were identified postdiscontinuation of contact precautions and both involved VRE isolates. One of these pairs shared a medical ward within the defined window (7 d before or after the index blood culture), while the other had no documented overlap in location. The third *E. faecium* pair, identified prediscontinuation, included one VRE (with vanA detected but not plasmid-associated) and one non-VRE isolate, and involved shared time in the operating suite. The *E. faecalis* pair, consisting of two non-VRE isolates, spanned both study periods and had no documented shared location.

### Plasmid transmission dynamics postdiscontinuation of contact precautions

**Figure 3.**
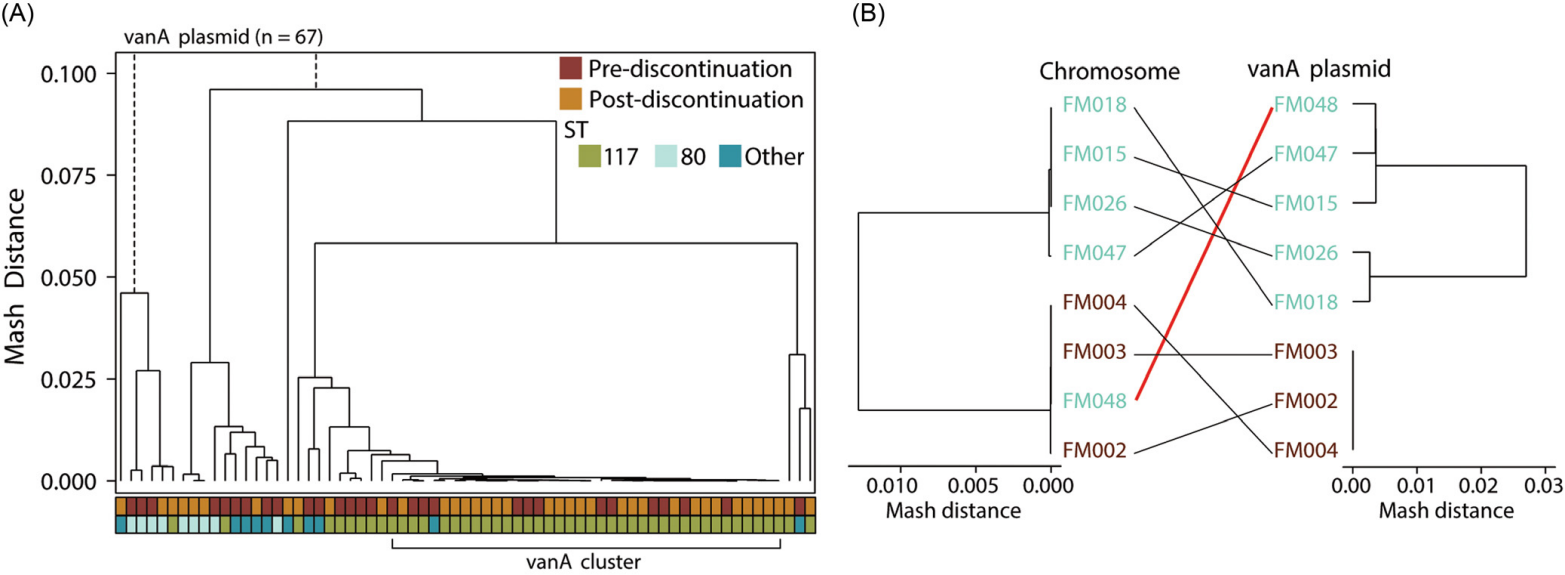
Dynamics of *vanA* plasmid among VRE blood culture isolates after discontinuation of contact precautions, Stanford Health Care, 2021–2023. Panel A displays hierarchical clustering of pairwise Mash distances calculated from whole-contig sketches for 67 vanA-containing contigs identified as plasmids by geNomad; beneath the dendrogram, two annotation tracks denote, from top to bottom, timing of obtention relative to discontinuation of contact precautions (Red Oxide = prediscontinuation; Golden Ocher = postdiscontinuation) and sequence type (ST) as defined by multilocus sequence typing (MLST, Moss Green = ST117, Pale Aqua = ST80, Teal Blue = other). Panel B illustrates a representative case of chromosomal–plasmid decoupling: Patient A (Turquoise Green) contributed five *E. faecium* isolates over 201 days (first four ST80; last isolate FM048 was ST117 and chromosomally grouped with Patient B [Chestnut Brown]), while its *vanA* plasmid remained closely related to Patient A’s earlier samples.

To assess whether plasmid-borne vancomycin resistance exhibited different transmission patterns than strain-level spread postdiscontinuation of contact precautions, we compared Mash distances among 67 *vanA*-containing plasmids from 53 patients. Of these, 66 were from *E. faecium* isolates. Several clusters of related *vanA* plasmids were identified (Figure 3A), including a dominant cluster of 38 highly related plasmids with Mash distance <0.001 (“low-diversity cluster”). There was no significant difference in the proportion of low-diversity cluster” plasmids identified pre-(14/29, 48.3%) and postdiscontinuation (24/38, 63.2%) of contact precautions (*P* = .223). We observed a case illustrating potential decoupling of the *vanA* plasmid and chromosomal evolution (Figure 3B). “Patient A” had five *E. faecium* isolates collected over 201 days. The last isolate was chromosomally most like isolates from “Patient B” and likely represented colonization by the dominant hospital ST117 *E. faecium* strain. However, the plasmid remained more closely related to earlier isolates from Patient A,” suggesting plasmid persistence despite chromosomal divergence or possible acquisition of a different strain.

### Molecular epidemiology of enterococci postdiscontinuation of contact precautions

**Figure 4.**
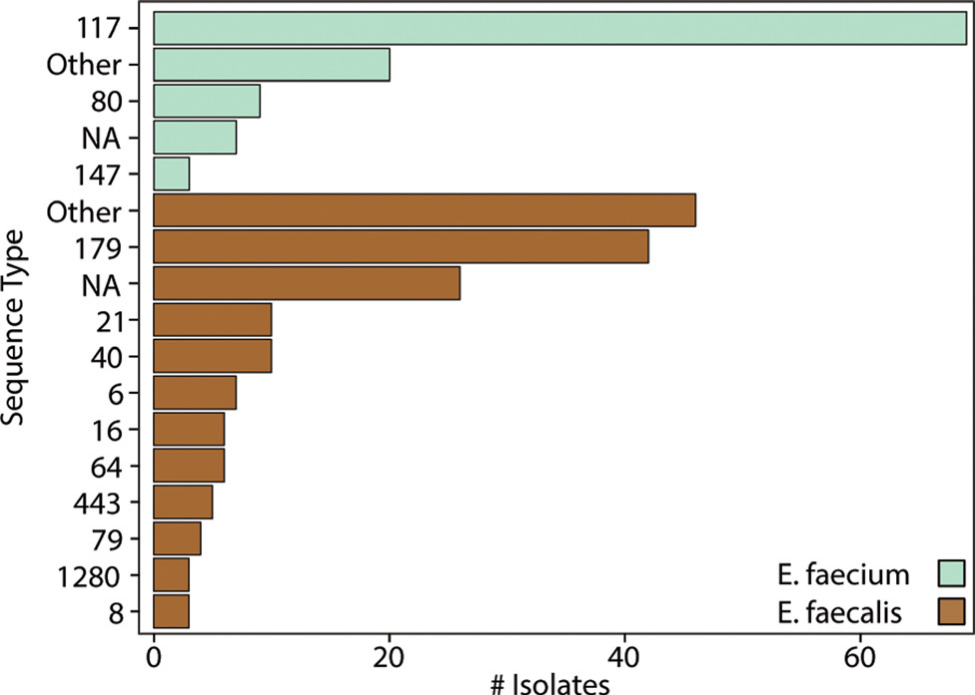
Distribution of sequence types among *E. faecium* and *E. faecalis* blood culture isolates, Stanford Health Care, 2021–2023. Bars show counts by sequence type; Seafoam Green represents *E. faecium*, Golden Brown represents *E. faecalis*. “Other” aggregates minor types; “NA” indicates undetermined sequence type.

Despite no clear evidence of increased patient-to-patient VRE strain or *vanA* plasmid transmission, we observed more *E. faecium* isolates of a vancomycin-resistant, *vanA*-positive sequence type postdiscontinuation of contact precautions (Figure 1A). The *E. faecium* population was dominated by ST117 (67 of 119 assemblies; Figure 4), which was frequently associated with *vanA* plasmid carriage and vancomycin resistance. A higher number of ST117 isolates were identified postdiscontinuation of contact precautions, but this was not statistically significant (pre-21/38 vs post-46/66, *P* = .138). By contrast, *E. faecalis* displayed more sequence type diversity, with ST179 accounting for 43 of 169 isolates and many falling outside the dominant lineage (Figure 4). Vancomycin resistance in *E. faecalis* was rare, with four isolates identified postdiscontinuation, only one of which carried a *vanA*-positive plasmid (Figure 1B).

Among the 68 ST117 isolates, nearly half (n = 28) were collected from two high-risk units: the surgical ICU (n = 14) and a transplant step-down unit (n = 14). Postdiscontinuation isolates (n = 49) were similarly concentrated in these units (surgical ICU, n = 13; transplant step-down unit, n = 11), with additional isolates from a medical ward (n = 4), the oncology-hematology ICU (n = 4), and the cardiothoracic ICU (n = 3). The remaining isolates postdiscontinuation were evenly distributed across units with a high volume of immunocompromised patients.

## Discussion

Stopping contact precautions for VRE infections was associated with no significant difference in potential transmission events among patients with bloodstream infections by classical epidemiology. Similarly, there was no increase in clonal transmission events based on bloodstream infection isolates. The proportion of highly related plasmids did not differ before or after discontinuation of contact precautions. However, the postdiscontinuation period was marked by a higher prevalence of the dominant *E. faecium* ST117 lineage, often carrying highly related vanA-positive plasmids.

Our observations are consistent with prior epidemiologic and genomic studies using short-read WGS that evaluated the impact of contact precautions on clonal VRE transmission. The STAR*ICU randomized controlled trial found no significant decrease in healthcare-associated VRE acquisition after the implementation of weekly screening and expanded use of contact precautions.^18^ A large quasi-experimental before-and-after study involving over 400 hospitals similarly reported no increase in VRE incidence following discontinuation of contact precautions.^7^ Biehl et al. conducted a multicenter cohort study across hematology-oncology wards in Germany and found confirmed patient-to-patient transmission rates of 9.4% and 5.6% at sites without and with contact precautions, respectively; antimicrobial exposure and host factors had a greater impact on VRE acquisition than contact precautions.^8^ Eichel et al. analyzed vancomycin-resistant *E. faecium* acquisition in ICUs before and after discontinuation of contact precautions and observed nosocomial transmission in 25% and 3.4% of bacteremia patients before and after discontinuation, respectively.^9^ Together, these studies and our own epidemiologic and genomic data suggest that contact precautions may have limited impact in controlling clonal patient-to-patient VRE transmission in acute-care hospitals.

Efforts focused solely on clonal spread may overlook the parallel role of MGEs in sustaining vancomycin resistance. MGEs play a key role in the dissemination and persistence of vancomycin resistance in *Enterococcus* species.^19^ The *vanA* gene is the dominant determinant in vancomycin-resistant *E. faecium* and *E. faecalis* globally.^1,20^ This gene is highly mobile, carried on the transposon Tn1546, which is frequently located on plasmids. This enables both transposition between genomic locations and horizontal transmission through plasmid dissemination.^19,21^ Previous efforts to characterize clonal transmission of *vanA*-carrying MGEs have relied on short-read WGS.^21^ However, such analyses are limited in reconstructing the nested architecture of resistance dissemination, particularly plasmids circulating across distinct bacterial lineages.

Long-read WGS increasingly enables reconstruction of complete plasmids involved in antimicrobial resistance transmission, including linear plasmids not easily resolved by short-read data alone. Recent reports from Japan have highlighted the role of such plasmids in the dissemination of *vanA*-mediated vancomycin resistance across multiple *Enterococcus spp*. Hashimoto et al. described a local outbreak involving *E. faecium*, *E. raffinosus*, and *E. casseliflavus* driven by interspecies transmission of a conjugative linear plasmid (pELF2) carrying *vanA*.^22^ Similarly, Fujiya et al. reported a prolonged multiclonal outbreak linked to a linear plasmid (pIHVA) that was horizontally transmitted among diverse *E. faecium* lineages and other *Enterococcus* spp.^23^ Both studies used hybrid sequencing approaches, combining short-and long-read data, along with conventional reference-based analyses and pulsed-field gel electrophoresis to characterize resistance spread. While these methods provided valuable insights, they also illustrate the limitations of earlier genomic tools in fully resolving plasmid diversity and transmission dynamics. We used long-read WGS and geNomad, a tool that classifies MGEs using genome architecture and machine learning.^15^ This allowed us to reconstruct highly-contiguous plasmid structures and better assess their movement independent of bacterial strains. We found no significant difference in the proportions of highly similar *vanA*-positive plasmids before and after the discontinuation of contact precautions. Furthermore, as shown in a case from our cohort, we observed the presumed independent horizontal transmission of a *vanA* plasmid between unrelated *E. faecium* strains.

Although proportions were not significantly different, we observed a numerically higher number of highly related *vanA*-positive plasmids following the discontinuation of contact precautions. Direct contact is widely regarded as the main route of transmission of resistant pathogens in acute-care hospitals.^24^ While direct contact remains the predominant assumed route of transmission for resistant pathogens in acute-care hospitals, this view may overstate the role of contact precautions in limiting resistance spread, particularly via MGEs. The perceived protection of contact precautions may lead to underestimating alternative transmission routes, including shared equipment, outpatient exposures, or even aerosols. First, portable medical equipment, such as computers on wheels, are frequently touched before patient contact but are not consistently included in standard disinfection practices.^25^ This equipment can serve as a reservoir for resistant bacterial strains and MGEs maintained by pathogenic or non-pathogenic bacteria and potentially transferred to patient microbiota^26^ Second, ambulatory and outpatient settings remain underrecognized in infection prevention efforts; shared spaces and rapid room turnover increase the likelihood of surface contamination.^27^ Pathogens have been isolated from high-touch surfaces in these settings, suggesting that MGEs may also persist in these environments.^28^ A third possibility involves the role of aerosols in MGE dissemination. Resistant bacterial strains can become aerosolized from sink drains and hospital plumbing systems,^29^ and MGEs may follow similar routes. Mobile genetic elements can account for up to 65% of extracellular, free-floating DNA in wastewater and have the potential to be transported by aerosols.^30^ Their detection in inhalable hospital particulate matter, along with evidence of conjugative transfer, suggests that MGEs may remain viable during aerosol transport and retain the ability to mediate horizontal gene transfer.^31^

Enterococci are widely distributed in nature, capable of behaving as commensals or as opportunistic pathogens, especially in immunocompromised patients.^32^ Both *E. faecium* and *E. faecalis* have become globally disseminated nosocomial pathogens. However, *E. faecium* includes a distinct hospital-adapted subpopulation rarely found outside healthcare settings. This distinction is less clear for *E. faecalis*, as strains causing infections in hospitals are also common in healthy individuals and animals.^4,32^ The majority of hospital-adapted *E. faecium* clones belong to clonal complex 17.^32,33^ They are characterized by the acquisition of adaptive genetic elements, including genes for metabolism, biofilm formation, and antibiotic resistance via the *vanA* gene.^32,34^ By contrast, *E. faecalis* generally lacks these adaptations; its virulence genes largely function as host-adaptive traits suited to a broad range of intestinal niches.^32^ Postdiscontinuation, *E. faecium* ST117 showed higher prevalence at Stanford Hospital overall, frequently carrying highly related *vanA*-positive plasmids and vancomycin resistance. By contrast, *E. faecalis* isolates displayed broader lineage diversity during the study period. *E. faecium* ST117 belongs to the healthcare-adapted clonal complex 17.^33^ This vancomycin-resistant lineage has been emerging globally, displacing earlier healthcare-associated strains due to its competitive advantages.^33,35–42^ This transition occurred largely after 2020,^33^ aligning with its emergence at Stanford University Hospital, although it was also detected in stool samples from bone marrow transplant patients at Stanford as early as 2015.^14^ This likely reflects both this broader global shift and the higher proportion of immunocompromised patients in the postdiscontinuation period, though a contribution from the discontinuation of contact precautions cannot be entirely excluded.

This study has several limitations. First, we included only bloodstream infection isolates, which underestimates the full burden of VRE colonization and transmission within the hospital but allowed us to explore the impact of discontinuing contact precautions on the most clinically significant transmission events. The absence of systematic colonization screening, such as rectal swabs, limited our ability to detect silent transmission events and may have reduced the sensitivity of our genomic surveillance. However, prior studies using short-read sequencing and evaluating acquisitions through colonization screening and bloodstream infections have not shown a major impact of contact precautions.^8,9^ Their effect on the silent transmission of MGEs remains to be studied. Second, while we used a conservative 20-SNP threshold informed by within-host comparisons, fixed cutoffs may not fully account for the varying evolutionary rates across *Enterococcus* lineages. However, this threshold has been used in prior genomic epidemiology studies^43^ and was additionally supported by empirical distributions observed in our dataset. Third, we lacked data on direct contact, shared healthcare providers, and environmental sources, which limited our ability to attribute transmission pathways with greater precision. Our findings of independent plasmid movement suggest that alternative transmission routes beyond direct patient contact should be further explored. Finally, generalizability may be limited, as our findings reflect the epidemiology of a single institution with a dominant, genomically stable ST117 clade, which may differ from settings with greater strain diversity or differing infection control practices. However, as a quaternary care transplant center, we provide data from a population with a high prevalence of immunocompromised patients, who have an increased burden of Enterococcus infection and are likely underrepresented in prior studies.

In conclusion, discontinuing contact precautions for VRE was not associated with increased enterococci transmission or *vanA* plasmid spread among bloodstream infection isolates. The postdiscontinuation period was marked by a higher prevalence of an already dominant *E. faecium* ST117 lineage among bloodstream infection isolates at Stanford Hospital. While vancomycin resistance spread remained limited regardless of contact precautions, the persistence of highly related *vanA*-positive plasmids, often carried by the hospital-adapted *E. faecium* ST117 lineage, suggests ongoing dissemination through alternative mechanisms. These findings call for a broader understanding of mobile genetic element transmission, as their mobility enables resistance persistence beyond direct patient contact and traditional inpatient settings.

## Supporting information

10.1017/ash.2026.10392.sm001 Rodriguez-Nava et al. supplementary material Rodriguez-Nava et al. supplementary material

## Data Availability

Long-read Enterococci genome assemblies generated in this study are available under NCBI BioProject ID PRJNA1236482.

## Supplementary material

The supplementary material for this article can be found at https://doi.org/10.1017/ash.2026.10392.

## References

[1] Kent AG , Spicer LM , Campbell D , et al. Sentinel surveillance reveals phylogenetic diversity and detection of linear plasmids harboring *vanA* and *optrA* among enterococci collected in the United States. Antimicrob Agents Chemother 2024;68:e00591–e00524. 10.1128/aac.00591-24. 39404260 PMC11539240

[2] Hawkins MR , Medvedeva N , Wang H , Banaei N , Holubar MK. “Keeping us on our toes”: a review of what clinicians need to know about vancomycin-variable Enterococcus. Antimicrob Steward Healthc Epidemiol 2024;4:e200. 10.1017/ash.2024.449. 39563924 PMC11574585

[3] Recommendations for preventing the spread of vancomycin resistance: recommendations of the Hospital Infection Control Practices Advisory Committee (HICPAC). Am J Infect Control 1995;23:87–94. 10.1016/0196-6553(95)90104-3. 7639408

[4] Hollenbeck BL , Rice LB. Intrinsic and acquired resistance mechanisms in enterococcus. Virulence 2012;3:421–569. 10.4161/viru.21282. 23076243 PMC3485979

[5] Fiore E , Van Tyne D , Gilmore MS. Pathogenicity of Enterococci. Microbiol Spectr 2019;7:7.4.9. 10.1128/microbiolspec.GPP3-0053-2018. PMC6629438 31298205

[6] Li S , Paras ML. Should contact precautions be used for patients with MRSA infection and colonization in acute care settings? NEJM Evid 2024;3:EVIDtt2300302. 10.1056/EVIDtt2300302. 38320491

[7] Martin EM , Colaianne B , Bridge C , et al. Discontinuing MRSA and VRE contact precautions: defining hospital characteristics and infection prevention practices predicting safe de-escalation. Infect Control Hosp Epidemiol 2022;43:1595–602. 10.1017/ice.2021.457. 34847970

[8] Biehl LM , Higgins PG , Stemler J , et al. Impact of single-room contact precautions on acquisition and transmission of vancomycin-resistant enterococci on haematological and oncological wards, multicentre cohort-study, Germany, January−December 2016. Euro Surveill 2022;27:2001876. 10.2807/1560-7917.ES.2022.27.2.2001876. 35027104 PMC8759111

[9] Eichel VM , Boutin S , Frank U , et al. Impact of discontinuing contact precautions and enforcement of basic hygiene measures on nosocomial vancomycin-resistant Enterococcus faecium transmission. J Hosp Infect 2022;121:120–127. 10.1016/j.jhin.2021.11.020. 34861314

[10] Kamathewatta KI , Bushell RN , Young ND , et al. Exploration of antibiotic resistance risks in a veterinary teaching hospital with Oxford nanopore long read sequencing. PLoS One 2019;14:e0217600. 10.1371/journal.pone.0217600. 31145757 PMC6542553

[11] Bonten MJ , Hayden MK , Nathan C , et al. Epidemiology of colonisation of patients and environment with vancomycin-resistant enterococci. Lancet 1996;348:1615–1619. 10.1016/S0140-6736(96)02331-8. 8961991

[12] Rubin IMC , Pedersen MS , Mollerup S , et al. Association between vancomycin-resistant Enterococcus faecium colonization and subsequent infection: a retrospective WGS study. J Antimicrob Chemother 2020;75:1712–1715. 10.1093/jac/dkaa074. 32125377

[13] Wammes LJ , Voor In ‘T Holt AF , Klaassen CHW , Vos MC , Verkaik NJ , Severin JA. Contact tracing for vancomycin-resistant Enterococcus faecium (VRE): evaluation of the Dutch policy of quintuple screening cultures. Eur J Clin Microbiol Infect Dis 2023;42:993–999. 10.1007/s10096-023-04632-7. 37351725 PMC10345005

[14] Grieshop MP , Behr AA , Bowden S , Lin JD , Molari M , Reynolds GZ , Brooks EF , Doyle B , Moore AA , Rodriguez-Nava G , Salinas JL , Banaei N, Bhatt AS . Transposable elements are driving rapid adaptation of *Enterococcus faecium*. Nature 2026. 10.1038/s41586-026-10373-2. PMC13216065 42020750

[15] Camargo AP , Roux S , Schulz F , et al. Identification of mobile genetic elements with geNomad. Nat Biotechnol 2024;42:1303–1312. 10.1038/s41587-023-01953-y. 37735266 PMC11324519

[16] Ondov BD , Treangen TJ , Melsted P , et al. Mash: fast genome and metagenome distance estimation using minHash. Genome Biol 2016;17:132. 10.1186/s13059-016-0997-x. 27323842 PMC4915045

[17] Harris SR. SKA: split kmer analysis toolkit for bacterial genomic epidemiology 2018. 10.1101/453142.

[18] Huskins WC , Murray P , Walker ME , Jernigan JA , Goldmann DA. Intervention to reduce transmission of resistant bacteria in intensive care. N Engl J Med 2011;364:1407–1418. 21488763 10.1056/NEJMoa1000373 PMC3410743

[19] Hegstad K , Mikalsen T , Coque TM , Werner G , Sundsfjord A. Mobile genetic elements and their contribution to the emergence of antimicrobial resistant Enterococcus faecalis and Enterococcus faecium. Clin Microbiol Infect 2010;16:541–554. 10.1111/j.1469-0691.2010.03226.x. 20569265

[20] Faron ML , Ledeboer NA , Buchan BW. Resistance mechanisms, epidemiology, and approaches to screening for vancomycin-resistant Enterococcus in the health care setting. J Clin Microbiol 2016;54:2436–2447. 10.1128/JCM.00211-16. 27147728 PMC5035425

[21] Arredondo-Alonso S , Top J , Corander J , Willems RJL , Schürch AC. Mode and dynamics of vanA-type vancomycin resistance dissemination in Dutch hospitals. Genome Med 2021;13:9. 10.1186/s13073-020-00825-3. 33472670 PMC7816424

[22] Hashimoto Y , Kita I , Suzuki M , Hirakawa H , Ohtaki H , Tomita H. First report of the local spread of vancomycin-resistant Enterococci ascribed to the interspecies transmission of a *vanA* gene cluster-carrying linear plasmid. mSphere 2020;5:e00102–e00120. 10.1128/mSphere.00102-20. 32269153 PMC7142295

[23] Fujiya Y , Harada T , Sugawara Y , et al. Transmission dynamics of a linear vanA-plasmid during a nosocomial multiclonal outbreak of vancomycin-resistant enterococci in a non-endemic area, Japan. Sci Rep 2021;11:14780. 10.1038/s41598-021-94213-5. 34285270 PMC8292306

[24] Wolfensberger A , Clack L , Kuster SP , et al. Transfer of pathogens to and from patients, healthcare providers, and medical devices during care activity—a systematic review and meta-analysis. Infect Control Hosp Epidemiol 2018;39:1093–1107. 10.1017/ice.2018.156. 30039774

[25] Jinadatha C , Villamaria FC , Coppin JD , et al. Interaction of healthcare worker hands and portable medical equipment: a sequence analysis to show potential transmission opportunities. BMC Infect Dis 2017;17:800. 10.1186/s12879-017-2895-6. 29281998 PMC5745722

[26] Von Wintersdorff CJH , Penders J , Van Niekerk JM , et al. Dissemination of antimicrobial resistance in microbial ecosystems through horizontal gene transfer. Front Microbiol 2016; 7:173. 10.3389/fmicb.2016.00173. 26925045 PMC4759269

[27] Siegel JD , Rhinehart E , Jackson M , Chiarello L. Guideline for isolation precautions: preventing transmission of infectious agents in health care settings. Am J Infect Control 2007;35:S65–164. 10.1016/j.ajic.2007.10.007. 18068815 PMC7119119

[28] Cadnum JL , Pearlmutter BS , Jencson AL , et al. Microbial bioburden of inpatient and outpatient areas beyond patient hospital rooms. Infect Control Hosp Epidemiol 2022;43:1017–1021. 10.1017/ice.2021.309. 34294185

[29] Goforth MP , Boone SA , Clark J , et al. Impacts of lid closure during toilet flushing and of toilet bowl cleaning on viral contamination of surfaces in United States restrooms. Am J Infect Control 2024;52:141–146. 10.1016/j.ajic.2023.11.020. 38276944

[30] Calderón-Franco D , Van Loosdrecht MCM , Abeel T , Weissbrodt DG. Free-floating extracellular DNA: systematic profiling of mobile genetic elements and antibiotic resistance from wastewater. Water Res 2021;189:116592. 10.1016/j.watres.2020.116592. 33171295

[31] Zhou Z-C , Shuai X-Y , Lin Z-J , Liu Y , Zhu L , Chen H. Prevalence of multi-resistant plasmids in hospital inhalable particulate matter (PM) and its impact on horizontal gene transfer. Environ Pollut 2021;270:116296. 10.1016/j.envpol.2020.116296. 33341549

[32] Guzman Prieto AM , Van Schaik W , Rogers MRC , et al. Global emergence and dissemination of enterococci as nosocomial pathogens: attack of the clones? Front Microbiol 2016;7:788. 10.3389/fmicb.2016.00788. 27303380 PMC4880559

[33] Mills EG , Hewlett K , Smith AB , et al. Bacteriocin production facilitates nosocomial emergence of vancomycin-resistant Enterococcus faecium. Nat Microbiol 2025;10:871–881. 10.1038/s41564-025-01958-0. 40119148 PMC11964922

[34] Palmer KL , Kos VN , Gilmore MS. Horizontal gene transfer and the genomics of enterococcal antibiotic resistance. Curr Opin Microbiol 2010;13:632–639. 10.1016/j.mib.2010.08.004. 20837397 PMC2955785

[35] Tedim AP , Lanza VF , Manrique M , et al. Complete genome sequences of isolates of Enterococcus faecium sequence type 117, a globally disseminated multidrug-resistant clone. Genome Announc 2017;5:e01553–e01516. 10.1128/genomeA.01553-16. 28360174 PMC5374248

[36] Weber A , Maechler F , Schwab F , Gastmeier P , Kola A. Increase of vancomycin-resistant Enterococcus faecium strain type ST117 CT71 at Charité - Universitätsmedizin Berlin, 2008 to 2018. Antimicrob Resist Infect Control 2020;9:109. 10.1186/s13756-020-00754-1. 32678047 PMC7364619

[37] Xanthopoulou K , Peter S , Tobys D , et al. Vancomycin-resistant Enterococcus faecium colonizing patients on hospital admission in Germany: prevalence and molecular epidemiology. J Antimicrob Chemother 2020;75:2743–2751. 10.1093/jac/dkaa271. 32699884

[38] Rodríguez-Lucas C , Fernández J , Raya C , et al. Establishment and persistence of glycopeptide-resistant *Enterococcus faecium* ST80 and ST117 clones within a health care facility located in a low-prevalence geographical region. Microb Drug Resist 2022;28:217–221. 10.1089/mdr.2021.0171. 34705570

[39] Santona A , Taviani E , Fiamma M , et al. Occult vancomycin-resistant Enterococcus faecium ST117 displaying a highly mutated vanB2 operon. Antibiotics 2023;12:476. 10.3390/antibiotics12030476. 36978343 PMC10044008

[40] Bezdicek M , Hanslikova J , Nykrynova M , et al. New multilocus sequence typing scheme for Enterococcus faecium based on whole genome sequencing data. Microbiol Spectr 2023;11:e05107–e05122. 10.1128/spectrum.05107-22. 37306567 PMC10434285

[41] Rath A , Kieninger B , Caplunik-Pratsch A , et al. Concerning emergence of a new vancomycin-resistant Enterococcus faecium strain ST1299/CT1903/vanA at a tertiary university centre in South Germany. J Hosp Infect 2024;143:25–32. 10.1016/j.jhin.2023.10.008. 37852539

[42] Hammerum AM , Karstensen KT , Roer L , et al. Surveillance of vancomycin-resistant enterococci reveals shift in dominating clusters from vanA to vanB Enterococcus faecium clusters, Denmark, 2015 to 2022. Euro Surveill 2024;29:2300633. 10.2807/1560-7917.ES.2024.29.23.2300633. 38847117 PMC11158013

[43] Sundermann AJ , Rangachar Srinivasa V , Mills EG , et al. Genomic sequencing surveillance of patients colonized with vancomycin-resistant *Enterococcus* (VRE) improves detection of hospital-associated transmission 2024. 10.1101/2024.05.01.24306710. PMC12351959 40804750

